# Macrophage Activation and Functions during Helminth Infection: Recent Advances from the Laboratory Mouse

**DOI:** 10.1155/2018/2790627

**Published:** 2018-07-02

**Authors:** Marion Rolot, Benjamin G. Dewals

**Affiliations:** Immunology-Vaccinology, Department of Infectious and Parasitic Diseases, Faculty of Veterinary Medicine-FARAH, University of Liège, Liège, Belgium

## Abstract

Macrophages are highly plastic innate immune cells that adopt an important diversity of phenotypes in response to environmental cues. Helminth infections induce strong type 2 cell-mediated immune responses, characterized among other things by production of high levels of interleukin- (IL-) 4 and IL-13. Alternative activation of macrophages by IL-4 *in vitro* was described as an opposite phenotype of classically activated macrophages, but the *in vivo* reality is much more complex. Their exact activation state as well as the role of these cells and associated molecules in type 2 immune responses remains to be fully understood. We can take advantage of a variety of helminth models available, each of which have their own feature including life cycle, site of infection, or pathological mechanisms influencing macrophage biology. Here, we reviewed the recent advances from the laboratory mouse about macrophage origin, polarization, activation, and effector functions during parasitic helminth infection.

## 1. Introduction

Parasitic helminths infect the majority of vertebrates [1]. Although parasitic helminths are near to absent in northwestern countries in humans, they are still responsible for infecting more than a quarter of the human population, essentially afflicting people who live in areas of poverty in the developing world [2], and they are also heavily present in domestic animals of veterinary importance [3]. In humans, 1.5 billion people are infected with soil-transmitted helminths (or intestinal nematodes) that persist in the intestine as adult worms for a prolonged period of time [4], filarial nematodes are tissue-dwelling parasites of more than 150 million people [5], while blood flukes (schistosomes) infect about 240 million people worldwide and induce chronic systemic and liver disease [6]. In addition, infection with larval stages of Taeniids remains an important zoonotic problem.

Helminths have evolved to adapt to the host they infect and developed immune evasion strategies that have in return shaped the immune system of the infected host. Such evolution may be explained by different phenomena, the most evident being that many helminths undertake specific multiorgan migratory trajectories before reaching their final destination such as the lung, intestine, liver, or blood vessels where they can persist and cause chronic infections. Helminths must also ensure that their offspring will find their way out without being stopped by the host immune system [7]. These often-complex life cycles have lead helminths to develop mechanisms to invade and migrate through the host while modulating the immune system and ensure their long-lasting persistence in their host [8]. As an example, the intestinal nematode *Heligmosomoides polygyrus* produces a TGF-*β* mimic during its invasive stages causing the induction of regulatory T cells (Tregs) in mice [9], a T cell subset that controls immunity in infection, allergy, and autoimmunity [10]. Besides, appropriate immune response is needed to repair tissue damage linked to parasite migration or to avoid damage caused by excessive immune activation. Therefore, immune modulatory mechanisms like induction of Tregs highlight the fact that these parasites are shaping the host immune system to reach a well-balanced tradeoff between immune evasion for parasite persistence and the modulation of host tissue damage to reduce as much as possible deleterious effects of worm persistence.

Parasitic helminths generally induce strong type 2 immunity that normally controls parasite infection and is characterized by production of type 2 cytokines like interleukin- (IL-) 4, IL-5, and IL-13 by innate cells (group 2 innate lymphoid cells (ILC2s), basophils, eosinophils, neutrophils, and macrophages) and CD4^+^ T helper 2 (Th2) lymphocytes. Type 2 cell-mediated immunity is a general feature of helminth infection regardless of the multivariate sites of colonization of the numerous helminth species [11] and is conserved from jawed fish to mammals [7]. Studies on mouse models of helminth infections have provided essential findings towards understanding type 2 immunity induction as well as its effector functions [12]. An important aspect about type 2 cell-mediated responses against parasitic helminths is that they are induced for controlling parasite infection but they also mediate the tolerance of parasite persistence [1]. In a number of cases, effector responses induced during type 2 immune responses promote the expulsion of intestinal helminths and prevent reinfection. Although type 2 immune responses developed in infected patients are not always sufficient to prevent disease, the induction of such responses aim to keeping parasite burdens under levels potentially resulting in pathologic sequelae (anaemia, growth retardation, fibrosis, etc.) that can be severely detrimental at the individual level and significantly delay socioeconomic development at the population level [13]. Besides promoting resistance to high-burden helminth infection, type 2 immune responses also include modulation/resolution of proinflammatory responses and tissue repair without directly affecting worm persistence [14]. Interestingly, Th2 cells in the liver ensure appropriate development of schistosome worms, further highlighting coevolution of the parasite and its host [15]. Thus, such tolerance mechanisms would permit low number of worms to persist while avoiding immunopathology. Coevolution of low-burden parasitic helminths with their respective host would be encouraged and could even lead, in some settings, to a mutually beneficial relationship of the host and parasite. Indeed, low-burden chronic infections with helminths are mostly asymptomatic and have demonstrated to be benefic to other diseases, especially in the case of autoimmunity and allergy [16, 17] as well as obesity or even autism [18], which advocates the use of specific helminths or derived products as therapeutic strategies while encouraging guided deworming campaigns [19].

Among type 2 cell-mediated mechanisms involved in the response against parasitic helminths, polarization of macrophages and their effector functions in host protection has been thoroughly studied. In this review, we will focus on the recent advances from the mouse model that lead our understanding on the roles of macrophages during parasitic helminth infection and discuss the challenges and opportunities in the future.

## 2. Macrophage Activation and Helminth Infection

Since their initial description by Élie Metchnikoff as immune cells mediating phagocytosis [20], we now know that macrophage function is not restricted to simply engulfing unicellular pathogens such as bacteria and protozoa but represents a large and heterogeneous family of cell subsets displaying different functions in physiological and pathological processes. Hence, macrophages are innate cells that can destroy pathogens but also clear apoptotic cell bodies and regulate the host immune response [21]. The functions of macrophages depend on their mode of activation that leads to their “polarization” towards effector functions. The activation of macrophages after infection by pathogens is mediated by pathogen-associated molecular patterns (PAMPs) and cytokines. For over 20 years, macrophages have been dichotomized in two main activation phenotypes that were essentially based on *in vitro* work on murine macrophages. Classically activated macrophages (CAMs, also known as M1) are instructed by bacterial products (LPS) and interferon- (IFN-) *γ* produced during type 1 immune responses and develop strong intracellular-killing nitric oxide (NO) in mice [21]. As opposed to classical macrophage activation, the work of Siamon Gordon in the 1990s highligted that macrophages could be alternatively activated by IL-4 [22] with increased mannose receptor (CD206), cellular responses associated with tissue repair, and reduced antimicrobial nitric oxide synthase (iNOS) production. Several molecules describing classical and alternative activation of macrophages in mice do not have an equivalent in humans. Recent work has aimed to provide a similar dichotomy in human macrophages [23–26]. Since the description of alternatively activated macrophages (AAMs, also known as M2), it now appears that macrophage activation cannot simply be subdivided in M1 and M2 subsets. Indeed, macrophages are not necessarily equals in terms of tissue origin or differentiation, and depending on the cytokine and toll-like receptor (TLR) agonists involved, these cells can exert an array of various levels of activation patterns going from two extremes: IFN-*γ*-activated [M (IFN-*γ*)] or IL-4-activated [M (IL-4)] macrophages [27]. Thus, defining classical versus alternative macrophage activation is not perfect to genuinely describe the complexity of polarization of the activation status of macrophage subsets in inflammatory and homeostatic settings at a given point in time and space. Nonetheless, efforts to further decode polarization in the era of single-cell sequencing and linking it to the actual functions of macrophage polarization are advancing [27–29].

**Figure 1:**
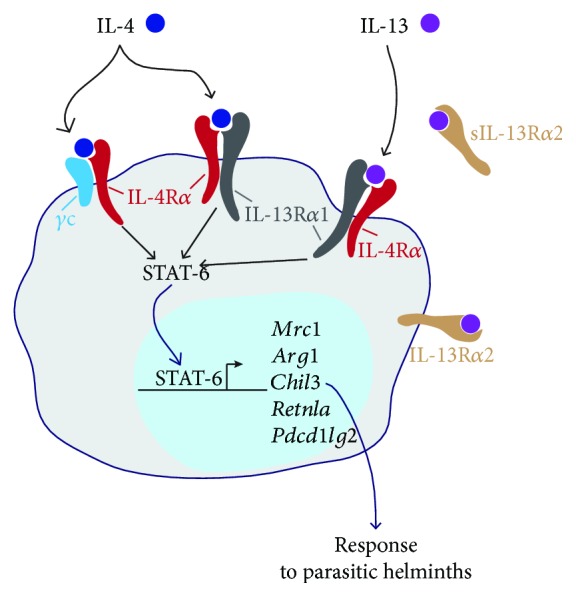
IL-4R*α*-dependent alternative macrophage activation during helminth infection. Type 2 innate and adaptive immune cells produce the cytokines IL-4 and IL-13 after exposure to parasitic helminths. In the laboratory mouse, these cytokines induce AAMs which are characterized by the upregulation of signature genes. IL-4R*α*: IL-4 receptor alpha chain; *γ*c: common gamma chain; IL-13R*α*1: IL-13 receptor alpha 1 chain; IL-13R*α*2: IL-13 receptor alpha 2 chain (sIL-13R*α*2, secreted form); STAT-6: signal transducer and activator of transcription 6; *Mrc1*: mannose receptor (CD206); *Arg1*: arginase 1; *Chil3*: chitinase-like 3 (Ym1); *Retnla*: resistin-like molecule alpha (Relm-*α*), *Pdcd1lg2*: programmed cell death 1 ligand 2 (PD-L2).

AAMs are defined by their response to IL-4 and/or IL-13, two cytokines that signal through the IL-4 receptor *α* chain (IL-4R*α*) (Figure 1). IL-4R*α* heterodimerizes with the common *γ*-chain (*γ*c) or IL-13R*α*1 to form the type I or type II receptor, respectively. Whereas IL-4 signals through both receptors, IL-13 only binds the type II receptor. Tissue colonization by helminths induces the rapid release of type 2 cytokines like IL-4 and IL-13 by ILC2s, eosinophils, neutrophils, basophils, or NKT cells (and at a later time point Th2 cells) that will instruct macrophages to adopt a phenotype close the AAM polarization observed by Gordon and Martinez [30]. In the rest of this review, we will refer to AAMs when describing helminth-induced IL-4/13-dependent activation of macrophages. Of note, additional cytokines have been shown to contribute to AAM polarization. IL-21 promotes the expression of IL-4R*α* and IL-13R*α*1 on macrophages [31], whereas IL-33 can signal through its T1/ST2 receptor on macrophages and drive an AAM phenotype together while triggering ILC2s and Th2 cells to promote IL-4 and IL-13 production [32, 33]. Besides, it has been convincingly reported that protective mechanism and tissue repair against *H. polygyrus* is further mediated by IgG-dependent induction of Arg1 in AAMs, independently of IL-4R*α* [34–36], and that surfactant protein D directly interacts with L4 larvae of *Nippostrongylus brasiliensis* and lung macrophages to promote their polarization into AAMs in the lung and contribute to controlling helminth infection [37]. Thus, activation of macrophages during helminth infection is not restricted to the simplified view of M (IL-4) polarized macrophages.

Tissue damage caused by helminth tissue colonization induces the release of alarmins such as IL-33 and IL-25 that leads to the activation of innate cells like ILC2s and the production of large amounts of IL-13, IL-5, and IL-4. Tissue alteration therefore indirectly leads to AAM polarization, and a recent report has shown that macrophages also need to directly respond to such alteration of the tissue by phosphatidylserin-mediated phagocytosis of apoptotic cells together with IL-4 and IL-13 signaling [38]. Interestingly, necroptosis in the liver after *Listeria monocytogenes* infection led to IL-4 release by basophils for AAM polarization of recruited monocytes in the liver for tissue repair [39].

The metabolism of arginine is a keystone that distinguishes AAMs from CAMs [21]. iNOS induced in CAMs after IFN-*γ* and bacterial PAMP activation metabolizes arginine to produce antimicrobial molecules like nitric oxide and citrulline. In AAMs, iNOS levels are reduced but arginase-1 (Arg1) expression increases. Arg1 hydrolyzes arginine in ornithine and urea. Because ornithine can be metabolized in proline (required for collagen deposition), AAM-derived Arg1 has long been regarded as essential for fibrosis and wound healing. However, work by Pesce and colleagues suggests rather that Arg1 in AAMs deplete arginine from the extracellular space for restraining T cell function by amino acid starvation than promoting fibrosis [40]. The latter hypothesis was further supported by the observation that hepatic stellate cells rather than Küpffer cells drive liver fibrosis after *Schistosoma mansoni* infection [41, 42]. Nonetheless, AAMs could still have healing capacities but not directly dependent on Arg1 activation [43, 44]. Instead, Esser-von Bieren and colleagues highlighted a role for ornithine in immobilizing *H. polygyrus* larvae after secondary infection [34]. Additional functions of AAM-dependent Arg1/ornithine may are still to be unraveled.

## 3. The Activation and Functions of AAMs after Infection with Helminths: Advances from Experimental Infection Models

### 3.1. Nematode Infections

Several groups have used nematode models in mice and demonstrated important functions of AAMs in helminth infection. In particular, studies involving intestinal *H. polygyrus* [45] and *N. brasiliensis* nematodes as well as filarial nematodes like *Brugia malayi* highlighted how induction of AAMs is determinant in the response against these parasites.

*H. polygyrus* (previously termed *Nematospiroides dubius*) is the dominant intestinal nematode of the European wood mouse [45], and its adaptation to the laboratory mouse has led to the suggestion to rename the adapted strain as *H. bakeri*. Nonetheless, additional data is still necessary to plainly justify the change in nomenclature [46]. *H. polygyrus* belongs to the order *Strongylida*, as the human hookworm parasites, and to the superfamily *Trichostrongyloidea*, as the ruminant parasites *Haemonchus contortus* and *Teladorsagia circumcincta*. *H. polygyrus* is an appropriate model of these chronic helminthiases, as primary infections can persist for many months in the intestine of susceptible strains of mice. Importantly, its life cycle is contained in the intestine which excludes potential confounding responses from the multiorgan migration observed for other nematode species, and its susceptibility to anthelminthic drugs like mebendazole renders studies on recall responses available. *H. polygyrus* infection induced AAMs in the intestine, and rapid resolution of reinfection with *H. polygyrus* has been shown to be dependent on Arg1 and intestinal AAMs [47, 48]. However, AAM induction after *H. polygyrus* infection resulted in increased susceptibility to bacterial infection with reduced bactericidal activities and colitis exacerbation [49]. In the same line, infection with *H. polygyrus* or treatment with IL-4 and anti-IFN-*γ* increased reactivation of a gammaherpesvirus from latently infected AAMs in C57BL/6 mice [50], suggesting alternative macrophage activation can have detrimental bystander effects.

**Figure 2:**
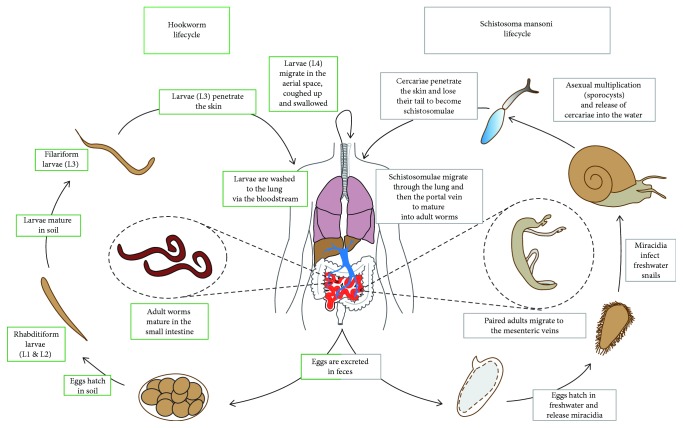
Graphical representation of the life cycles of hookworms (human: *Necator americanus* or *Ancylostoma duodenale*; mouse: *Nippostrongylus brasiliensis*) and *Schistosoma mansoni*.

*N. brasiliensis* (previously termed *Heligmosomum muris* and *Nippostrongylus muris*) is a nematode that naturally infects rats. Like *H. polygyrus*, *N. brasiliensis* belongs to the order *Strongylida*, as the human hookworm parasites *Necator americanus* or *Ancylostoma duodenale*. Albeit not directly related phylogenetically to human hookworms, *N. brasiliensis* has a very similar life cycle and is extensively used to investigate the host response to infection in the laboratory mouse. Free-living larvae invade the host percutaneously and enter the circulatory system, from where the larvae reach the lungs. Here, worms molt and breach the alveoli to reach the airway where they are coughed up, swallowed, and mature into adult worms in the intestinal lumen [51] (Figure 2). Infection of laboratory Balb/c or C57BL/6 mice induces strong type 2 cell-mediated responses that result in clearance of intestinal worms within 6–9 days after infection. IL-4 and IL-13 production after *N. brasiliensis* colonization of the host induces AAMs in the lung and the intestine [52, 53]. Intestinal macrophages after *N. brasiliensis* infection upregulate signature proteins of AAMs and depletion of intestinal macrophages by clodronate liposome treatment resulted in impaired expulsion of the nematode and affected smooth muscle contractility, partially involving Arg1 [53]. Thus, AAMs could contribute to the movement of luminal worms along the intestine collectively referred to as the “weep and sweep” mechanism, with increased intestinal contractility and mucous production. Whether or not the AAM presence seems to be essential for parasite clearance, their activation via the IL-4R*α* is not essential for resolving infection as observed in conditional knockout mice in macrophages for this receptor [54]. More work should focus on intestinal macrophages in parasite clearance to better define whether their role in promoting the weep and sweep is due to their alternative activation or whether it is just their presence as macrophages independently of their activation by IL-4 and IL-13.

*N. brasiliensis* larvae migrate through the lung and cause severe pulmonary pathology to the infected host, with hemorrhages that quickly resolve. Passage of larvae through the lung can result in a pathology initially analogous to allergic asthma that later develops in an emphysema-like disease [55–57]. Even though *N. brasiliensis* is responsible for fundamental changes in lung macrophage phenotypes with signatures of AAMs [52], a direct effect of lung AAMs on the regulation of parasite control remains unclear [58]. However, lung macrophages have been shown to contribute to resolving IL-17-driven inflammation and tissue damage in the lung after *N. brasiliensis* infection [59], and neutrophil response to the parasite appears to condition long-lived macrophages in the lung for controlling the nematode infection [60]. These results indicate that AAMs are key components to mediate control parasite infection in the lung and tissue repair, but it remains uncertain whether alternative activation *via* signaling through IL-4R*α* is truly essential.

Mouse infection with *Brugia malayi* (a filarial nematode that belongs to the order *Spirurida*, superfamily *Filarioidea*) also induces AAMs that strongly suppress T cell proliferation and have tissue repair functions [61, 62]. *B. malayi*-induced AAMs could be reprogrammed to kill bacteria [63] and displayed elevated gene expression for arachidonic acid metabolism pathways, resulting in increased levels of PGI2 and PPAR*γ*-mediated activation [64]. Recent data further showed that AAMs induced after *B. malayi* infection sustained eosinophil immunity via CCR3 [65]. Macrophage activation after *B. malayi* infection was controlled by the induction via IL-4 of the microRNA miR-378-3p that downregulated the PI3K/Akt-signaling pathway [66], illustrating a self-control mechanism that limits AAM activation and expansion during type 2 inflammatory settings.

Overall, we can conclude that while macrophages are key players in the clearance of nematodes from the infected host, signaling through IL-4R*α* is also essential for resistance to nematode infection *via* induction of type 2 cell-mediated immune responses but not necessarily through induction of AAMs.

### 3.2. Trematode Infections

A few studies investigated on the role of AAMs after liver fluke (*Fasciola hepatica*) infection [67, 68]. Although little is known on the role of AAMs during *F. hepatica* infection, a recent study showed that macrophage PD-L2 regulates type 1 immunity after infection [69], suggesting AAMs have regulatory functions.

The functions of AAMs after parasitic helminth infection have extensively been studied in mouse models of schistosomiasis—also known as bilharzia. *Schistosoma mansoni*, *S. haematobium*, and *S. japonicum* are the three main parasitic helminths responsible for human schistosomiasis and belong to the class *Trematoda* and the subfamily *Schistosomatidae*. Whereas *S. mansoni* and *S. haematobium* are found in Africa and the Middle East, *S. mansoni* is also found in Central and South America. *S. japonicum* infects people living in Asia, mainly in China and the Philippines [70]. Additional species can also be found in more localized areas. While *S. mansoni*, *S. japonicum*, *S. mekongi*, and *S. guineensis* are responsible for intestinal schistosomiasis, *S. haematobium* causes urogenital disease. The life cycle of the different species of schistosomes is similar and involves an intermediate freshwater snail host. The adult worms (males and females) live in the mesenteric veins of the infected host, where they mate and produce eggs. Each female worm can produce between 100 and 300 eggs per day and about half of them are excreted through faeces (*S. mansoni* and *S. japonicum*) or urine (*S. haematobium*). The other half of the produced eggs are trapped in host tissues such as the liver, intestine, or bladder, where they induce inflammation. Inflammation and tissue damage and remodeling elicited in reaction to parasite eggs are responsible for the main clinical signs including diarrhea, hematochezia, hepatomegaly, splenomegaly, ascite, or hematuria [70]. The eggs that reach freshwater will hatch and release free-living miracidia that infect a suitable intermediate snail host (*Biomphalaria* sp., *Bulinus* sp., or *Oncomelania* sp.). In the snail, the parasite then undergoes asexual replication through sporocyst stages during 4 to 6 weeks before shedding thousands of infectious cercariae into the water. The cercariae infect their mammalian host percutaneously, lose their tail, and migrate via the blood circulation to the lung where the maturing schistosomulae will become an adult worm that migrates to the liver veins and mesenteric venules. The parasite needs about 4–6 weeks before becoming mature adults and producing eggs (Figure 2).

Migration of the parasite through the host induces an immune reaction that differs depending on the stage of the life cycle, but despite repeated exposure, the immune response is not effective to prevent reinfection or clear parasites from the host. The direct consequence in areas where the prevalence of the parasite is high is the development of chronic disease.

*S. mansoni* infection of the laboratory mouse is well described and used as a model for human pathology [71, 72]. During the first weeks of infection, adult worms elicit an IFN-*γ*-dominated response. This response is then modulated by the arrival of the parasite eggs provoking a strong but not exclusive type 2 cell-mediated response that peaks around 7 to 8 weeks postinfection (“acute” phase) before being downregulated during the “chronic” phase by week 12 [73–76]. Type 2-dominated responses orchestrate the dynamic of the formation and maturation of inflammatory granulomas that is essential for protecting the host cells such as hepatocytes from cytotoxins produced by the eggs during the acute phase and then to heal the scar left by the dying eggs in the chronic phase [77]. These granulomas are mainly composed of CD4^+^ T lymphocytes, eosinophils, and AAMs [77]. Although macrophages and more particularly AAMs may play important roles in the induction of effective granulomatous response, much is needed to fully decipher their implication in the protective immune response against schistosomiasis [41, 78]. Unlike in nematode infection, protection against *S. mansoni* infection is not assessed by the efficiency of parasite expulsion. Indeed, susceptibility to *S. mansoni* is directly dependent on the host's ability to control egg-induced inflammation rather than directly controlling the number of blood-dwelling adult worms [40, 79, 80]. Current understanding of the control of egg-induced inflammation depends on the acute or chronic phase of infection.

Metabolically active and harmful eggs are retained in the host tissue during the acute phase and induce a granulomatous inflammatory response to wall them off from the host tissue. Early studies have shown that type 2 immunity is mainly protective against murine schistosomiasis during the acute phase, corresponding to the peak of egg production. Indeed, mice deficient for IL-4, IL-13, IL-4/13, IL-4/10, or IL-4R*α* all develop severe intestinal and liver pathology to acute *S. mansoni* infection and rapidly die of the infection [54, 79, 81–85]. In 2004, Herbert and collaborators studied *S. mansoni* infection in *Lyz2^cre^Il4ra^−/lox^* mice, knockdown for the IL-4R*α* chain specifically in lysozyme M-expressing macrophages and neutrophils [54]. The authors reported that *Lyz2^cre^Il4ra^−/lox^* mice suffered an acute wasting disease similar to *Il4ra^−/−^* mice, with evidence of hepatotoxicity and endotoxemia. Although these results suggested a central role of AAMs in the susceptibility to acute schistosomiasis, it may not be so clear. Conflicting results using low and high doses of infection recently rather suggested that *Lyz2^cre^Il4ra^−/lox^* mice are not highly susceptible to *S. mansoni* infection [80]. Indeed, Vannella and colleagues highlighted an incomplete deletion of IL-4R*α* among the heterogeneous macrophage populations of *S. mansoni*-infected *Lyz2^cre^Il4ra^−/lox^* mice. They observed an insufficient expression of *Lyz2* (encoding lysozyme M), therefore of cre-recombinase, in newly recruited, immature F4/80^hi^CD11b^hi^ macrophages which retained features of alternative activation. Interestingly, these data further provide an alternate explanation to the presence of IL-10-dependent Ym1- and CD206-expressing macrophages in the granulomas of *Lyz2^cre^Il4ra^−/lox^* mice [86]. Moreover, two recent studies demonstrated that AAMs in the liver after *S. mansoni* infection resulted from the maturation of recruited Ly6C^hi^ monocytes [87, 88]. Thus, AAMs' protective roles during acute schistosomiasis and control of intestinal permeability remain unclear and further urge the development of new tools to investigate the functions of AAMs during in schistosomiasis.  Table 1 summarizes the tools currently used or that could help understand macrophage functions in this context. As mentioned in the introduction, identification over the course of the infection of the range of macrophage polarizations in the liver and intestine should lead us towards novel mechanisms to better understand the true implication of AAMs in protection against egg-induced inflammation in acute schistosomiasis.

Liver fibrosis develops in the chronic phase of schistosomiasis. Although protective during the acute phase of the infection, type 2 inflammation can be detrimental during the chronic phase of infection with larger liver granulomas and increased collagen deposition, leading to portal hypertension, portosystemic venous shunts, and gastrointestinal hemorrhages. Using mice deficient for IL-13R*α*1 (type II receptor) or secreted decoy receptor IL-13R*α*2, it became clear that excess IL-13 is directly responsible for induction of collagen deposition and fibrosis, whereas both IL-4 and IL-13 mediate the inflammatory phenotype of egg-induced granulomas. Indeed, despite elevated levels of AAM gene signature expression in the liver, reduced fibrosis developed in *Il13ra1^−/−^* mice in response to *S. mansoni* infection [89]. In addition, neutralization of IL-13 by injection of a decoy receptor (sIL-13R*α*2) attenuated *S. mansoni*-induced liver fibrosis to a greater extent than suppression of IL-4 [90]. As mentioned above, although initially thought to promote collagen deposition via elevated levels of Arg1 expression, Pesce and collaborators showed that AAM-specific Arg1 reduces fibrosis rather than promotes it [40]. Using *Lyz2^cre^Arg1*^*lox/*lox^ or Tie2*^cre^Arg1*^*lox/*lox^ mice, they observed elevated fibrosis, increased granuloma volumes, and lower survival rate in the chronic phase of schistosomiasis. Interestingly, the idea that AAMs modulate type 2 immunity and fibrosis during chronic schistosomiasis was further supported by studies using mice deficient for resistin-like molecule *α* (Relm-*α*) that developed increased type 2 immune responses as well as increased fibrosis and hepatosplenic pathology [58]. To add a level of complexity, increased granulomatous inflammation but no impact on the levels of fibrosis were observed in *Lyz2^cre^Il4ra^−/lox^* mice during the chronic stage of infection suggesting that there might be distinct subsets of AAMs in the liver after infection, as suggested above [80].

Overall, although their roles in tissue repair and survival during the acute phase of *S. mansoni* infection have yet to be clarified, AAMs or AAM-associated molecules were shown to be implicated in the control of excessive inflammation and in wound healing through regulation of IL-13-induced fibrosis.

### 3.3. Cestode Infections

The class *Cestoda* includes important zoonotic parasites of the family *Taeniidae* like *Echinococcus* sp. or *Taenia* sp. These segmented worms are characterized by an indirect life cycle with production of encysted larvae (metacestode) in intermediate host tissues and transmission to the final host *via* feeding on infected tissues. *T. solium*, *E. granulosus*, and *E. multilocularis* were ranked as the top 3 food-borne parasites based on multiple criteria including incidence, disease severity, or trade relevance and are therefore subject to both human and veterinary medical challenges [91]. Unlike other helminths, cestode infections remain an important concern in developed countries, probably due to the important public health risk and difficulty of treatment. Furthermore, prevalence of *E. multilocularis* reaches more than 10% in foxes of the most affected European countries like Estonia, Latvia, Lithuania, France, Switzerland, or Germany [92]. Associated pathologies are mostly linked to larval cystic stages inducing loss of function of the organ involved. Mice model for cestode infections includes intraperitoneal or intracranial injection of metacestodes from *Mesocestoides corti* [93–95] or *T. crassiceps* [95] (both related to *T. solium*), injection of protoscoleces extracted from *E. multilocularis* or *E. granulosus* hydatid cyst [96], or infection with eggs or metacestodes from *Hymenolepis diminuta* and *H. nana* [97, 98]. The particularity of some *Taeniid* metacestodes to reproduce asexually is used in biological models. For instance, *T. crassiceps* larvae injected into the peritoneal cavity cause long-lasting infection and reproduce through outward budding. Nonetheless, metacestodes are usually sessile and grow in a fixed tissue site once established, leading to the development of various form of cysts depending on the species. The development of granulomatous immune responses against larval stages of cestode infections has been recently and extensively reviewed [99].

The role of macrophages in cestode infection is understudied, but interesting information comes from work on *T. crassiceps*. After *T. crassiceps* infection, an early type 1 immune response at the site of infection is shifted to a mix type 1/type 2 response with production of both IFN-*γ* and IL-4 [100, 101]. Control of *T. crassiceps* infection is strikingly different from that of other nematodes or trematodes with initial protective type 1 immune responses that control larval growth [102–104]. In particular, CAMs are suggested to control *T. crassiceps* infection through NO production. Indeed, blocking of NO synthase in susceptible wild-type BALB/c or resistant *Stat6^−/−^* mice resulted in increased parasites loads [105]. Further supporting a role for CAMs in the control of *T. crassiceps* infection, mice lacking migration inhibitory factor (MIF) were highly susceptible despite similar IFN-*γ* levels. Peritoneal macrophages of *MIF^−/−^* mice failed to respond to LPS and IFN-*γ* stimuli ex vivo and produce low levels of CAM-associated molecules like IL-12, TNF-*α*, or NO [106]. However, experiments using intraperitoneal or intracranial injection of *M. corti* metacestodes suggested a protective role of type 2 immune responses, with *Il4^−/−^* or *Stat6^−/−^* mice being highly susceptible and dying after infection [107, 108]. In addition, a number of studies of various cestode infections suggested that AAMs were mostly associated with immunoregulatory functions to avoid deleterious inflammation to tissues surrounding encysted metacestodes [95, 109–111]. But AAM presence and overexpression of AAM-associated molecules like PD-L1 and PD-L2 were also associated with increased susceptibility to infection with *T. crassiceps* [112, 113]. Thus, macrophages are present around the developing metacestodes and contribute to the granuloma formation [99], but the role of type 2 immune responses and in particular AAMs in this context remains to be fully elucidated.

### 3.4. IL-4-Driven Macrophage Proliferation and the Origin of AAMs after Helminth Infection

To understand the contribution of AAMs in helminth infection, understanding their origin is essential. For decades, the established dogma had been that tissue-resident macrophages were derived from circulating monocytes originating from the bone marrow. Challenging this idea, recent reports using fate-mapping experiments brought evidence that resident macrophages are established in the various tissues, including serous cavities, during organogenesis and shortly after birth from yolk sac and fetal liver precursors [114, 115]. Once established in their tissue niche, resident macrophages are usually characterized by their ability to self-renew by homeostatic proliferation. However, the capacity of resident macrophages to maintain in a long term without contribution of blood monocytes appears to be highly tissue-dependent. Evidence indicates their progressive replacement by bone-marrow-derived monocytes in specific tissues like the heart, pancreas, peritoneal and pleural cavities, skin, or intestine, while strain or sex might be important confounding factors [116–118]. Furthermore, monocyte-derived resident macrophages were shown to be able to adopt a phenotypical and transcriptional profile very close to that of embryonic resident macrophages, including the ability to self-renew through proliferation [119]. Similarly, during inflammatory processes, the expansion of macrophage populations is mostly associated with recruitment on monocytes but can also result from local proliferation of resident cells. Indeed, when initially attempting to deplete macrophages to investigate their role during mouse infection with the filarial nematode *Litomosoides sigmodontis* by clodronate treatment, Jenkins and colleagues found that tissue-resident macrophages were directly responding to IL-4 that induced their proliferation beyond homeostatic levels induced by CSF-1 [120, 121]. Local macrophage proliferation turned out to be not restricted to the pleura but also apparent in the liver, small intestine lamina propria, and peritoneal cavity after *H. polygyrus* infection whereas treatment with IL-4 complexes was sufficient to induce proliferation [122]. In addition, recent data highlighted the existence of an amplification system in addition to IL-4 that is needed for type 2 immunity within distinct tissues. *N. brasiliensis* migration through the lung induced IL-4R*α*-dependent production of surfactant protein- (SP-) A by alveolar epithelial cells. Macrophages from *SP-A^−/−^* mice infected with *N. brasiliensis* or treated with IL-4c failed to proliferate and to upregulate AAM markers [123]. Consistent with a direct role of SP-A on macrophages to boost IL-4-mediated macrophages activation, *in vitro* treatment with SP-A and IL-4 increased proliferation and upregulation of AAM markers in WT but not *Il4ra^−/−^* alveolar macrophages, compared to IL-4 treatment alone [123]. Similarly, SP-D was also shown to interact with alveolar macrophages after *N. brasiliensis* infection and to increase expression of AAM markers after IL-4/IL-13 treatment ex vivo [37]. Interestingly, the complement factor C1q, but not SP-A, was shown to have a similar effect on peritoneal macrophages and in the liver while alveolar macrophages were not responsive to C1q [123]. Thus, IL-4 induces production of SP-A and C1q and the expression of their receptor, myosin 18A, suggesting the existence within different tissues of an amplification system for local type 2 immune responses [123]. In addition to IL-4, IL-33 has also been shown to mediate macrophage proliferation through a distinct mechanism [124].

Local amplification is not the unique source of the AAM response after helminth infection. Indeed, egg-induced inflammation after *S. mansoni* infection causes the development of AAM-rich liver granulomas. As opposed to that observed with other helminth models, *S. mansoni* egg deposition is associated with a recruitment of Ly6C^hi^ monocytes of bone-marrow origin and these inflammatory monocytes differentiate into macrophages [87]. Although Küpffer cells (liver-resident macrophages) do respond to the infection and seem to proliferate at low levels, the main response rather seemed to be due to monocyte recruitment and differentiation into AAMs [88]. Although it remains unclear whether signaling through IL-4R*α* is involved in the recruitment and responses of recruited monocytes, AAMs derived from infiltrating monocytes or tissue-resident macrophage proliferation have distinct phenotypes, with monocyte-derived AAMs being associated with immune regulation properties [117, 125]. AAM origin could therefore influence their contribution to pathology as observed after bleomycine-induced lung fibrosis [126] or protection against pathogens [117]. Interestingly, recruited monocytes to the liver after *S. mansoni* infection acquire a phenotype of liver resident macrophages after alternative activation that is dependent on vitamin A [127]. Whether these macrophages of bone-marrow origin in the liver take over the niche of Küpffer cells as observed in diphtheria toxin-treated Clec4f-DTR mice or Küpffer cell necroptosis caused by *L. monocytogenes* infection is unknown [39, 119], like it is also unknown what signal(s) induce(s) monocyte recruitment to the liver where *S. mansoni* eggs are responsible for elevated IL-4 and IL-13 responses.

### 3.5. Effector Molecules

Following parasitic helminth infection, IL-4/13 signaling in AAMs results in the activation of the transcription factor signal transducer and activator of transcription (STAT) 6 [128]. Upon activation in macrophages, STAT-6 binds to promotor regions of multiple genes and promotes their expression that results in AAM polarization and proliferation. Of note, STAT-6 also promotes the expression to other transcription factors such as peroxisome proliferator-activated receptor- (PPAR-) *γ*, Krüppel-like factor (KLF) 4, or interferon regulatory factor (IRF) 4. Upon polarization, AAMs upregulate the gene expression of a range of signature proteins including the mannose receptor (CD206), Arg1, chitinases and chitinase-like molecules, resistin-like molecules, or programmed cell death ligand 2 (Figure 1). We describe below the understanding of effector molecules produced by AAMs in the laboratory mouse, but as specified before, some of these molecules either are not reliable markers or do not have orthologues in humans. Nonetheless, investigating their functions in mice is important to understand the full spectrum of how AAMs function in different species, including humans.

Interactions between helminths and C-type lectins such as the mannose receptor (CD206, encoded by *Mrc1*) or the macrophage galactose-type C-type lectin 2 (MGL2 or CD301b) are understudied [129, 130]. CD206 is upregulated on macrophages upon treatment with IL-4, IL-13, and IL-10 [22, 131] as well as prostaglandins PGE1 and PGE2 [130]. Although CD206 has extensively been used in the characterization of AAMs induced upon parasitic helminth infection [21, 75, 86], its exact role remains uncertain. Helminths secrete large amounts of highly glycosylated proteins that can bind to CD206-expressing immune cells such as macrophages or dendritic cells. Excretory/secretory products of schistosomules from *S. mansoni* limit the production of proinflammatory cytokines [132–134]. *S. mansoni* omega-1, a major secreted egg glycoprotein responsible for the induction of type 2 immune responses [135, 136], is mainly internalized by CD206-expressing dendritic cells and therefore impairs protein synthesis through its RNAse activity [137]. In addition to *S. mansoni*, CD206 has been shown to bind other helminth species like *T. spiralis* muscle larvae [138], *T. muris* excretory/secretory products [139], or *F. hepatica* tegumental proteins [140]. Nonetheless, the role of AAM-specific CD206 expression remains elusive during helminth infection, although CD206 seems to be a relevant surface marker for monocyte-derived AAMs [127]. The macrophage galactose-type C-type lectin 2 (MGL2 or CD301b) is another C-type lectin upregulated in AAMs in response to IL-4 and IL-1 or helminth trigger [129]. While CD301b^+^ dendritic cells have been shown to be essential for the induction of optimal type 2 immune response during OVA immunization or helminth infection [141, 142], CD301b-expressing macrophages appeared to play an important role in wound healing [143].

Arg1 is one of the two isoforms of arginase enzymes that is mainly associated with AAMs. Arg1 is constitutively expressed in the liver, but AAM-specific Arg1 has been shown to control excessive fibrosis *via* modulation of CD4^+^ T cell proliferation after chronic parasitic helminth infection has been highlighted using *Lyz2^cre^Arg1*^*lox/*lox^ or *Tie2^cre^Arg1*^*lox/*lox^ mice [40], as described above. In addition, bone-marrow chimeras using *Arg1^−/−^* hematopoietic cells revealed the important role of bone-marrow-derived Arg1 in controlling intestinal inflammation caused by *S. mansoni* eggs by suppressing IL-12/IL-23p40 production and maintaining the Treg/Th17 balance within the intestinal mucosa [144]. Nonetheless, the role of Arg1 might depend on the helminth species as macrophage-specific expression of Arg1 was dispensable for *T. muris* expulsion whereas it has been involved in promoting the expulsion of *H. polygyrus* and *N. brasiliensis* [48, 53]. The role of Arg1 further seems to depend on the tissue, as attested by the observation that although lung macrophages are essential to maintain IL-13-dependent inflammation and fibrosis [145], macrophage-specific Arg1 is largely dispensable to control helminth-driven lung inflammation [146].

Chitinases are molecules that cleave chitin, a widespread biopolymer of N-acetyl-beta-D-glucosamine that provides structural rigidity to fungi, arthropods, and helminths. In addition to the acidic mammalian chitinase (AMCase), upregulation of chitinase-like molecule expression such as Ym1 (encoded by *Chil3*), Ym2 (encoded by *Chil4*), or BRP-39 (encoded by *Chil1*) has been associated with AAMs [64, 122, 147, 148]. As opposed to chitinases, chitinase-like molecules do not have chitinase activity. A number of studies have focused on the role of AMCase in allergic lung disease, as its expression is increased in epithelial cells and macrophages in IL-4-dominated inflammation in the lung and intestinal tract. However, it recently turned out that AMCase is largely dispensable for allergic responses in the lung while essential for priming protective effector responses against parasitic nematodes *H. polygyrus* and *N. brasiliensis* [149]. Whether AAMs could be a source of AMCase after helminth infection remains to be determined. Ym1 and Ym2 do not have human orthologs, which explains why the role of these proteins has been disregarded even though Ym1 is strongly upregulated in AAMs and has been shown to attract eosinophils [150]. Mouse BRP-39 however is an ortholog of the human YKL-40 (encoded by *CHI3L1*), and an elegant study using neutralizing anti-Ym1 antibody treatment or *Chil1^−/−^* mice suggested a role of Ym1 and chitinase-like molecules in inducing IL-17 secretion by *γδ*-T cells to promote neutrophil recruitment in the lung after infection with *N. brasiliensis* [151]. Although nematode-induced Ym1 results in IL-17 responses and lung injury, AAMs can control IL-17-driven inflammation in the lung [59]. Moreover, lung neutrophil infiltration after *N. brasiliensis* infection crosstalk and regulate lung AAMs for rapid worm killing [60].

Resistin-like molecule-*α* (Relm-*α*, encoded by *Retnla*), otherwise known as “found in inflammatory zone 1” (Fizz1), is a member of a family of cysteine-rich secreted proteins that make important contributions to host control of helminth infections [152]. Relm-*α* is upregulated in AAMs and also in eosinophils and epithelial cells and is predominantly found in the lungs upon type 2 inflammation, such as allergic inflammation or exposure to parasitic helminths [153]. *Retnla^−/−^* mice displayed increased type 2 cell-mediated cytokine responses with elevated IL-4, IL-13, and IL-5 production by CD4^+^ T cells [58, 154]. Relm-*α* was shown to directly bind to CD4^+^ T cells and inhibit type 2 cytokine production, with no effect on T cell activation or proliferation [154]. During *S. mansoni* egg-induced inflammation, Relm-*α* is also expressed by epithelial cells and eosinophils, but coculture experiments showed that Relm-*α*-dependent inhibition of type 2 cytokine production can be supported by macrophages [154]. However, in the liver of *S. mansoni*-infected mice, eosinophils appear to be the main, if not the exclusive, source of Relm-*α* [58]. In addition, Relm-*α* seems to be dominating over intestinal Relm-*β* in the control of *N. brasiliensis* [155]. There exists a strict dependency of Relm-*α* expression on type 2 cytokines as mice deficient for IL-4R*α*, IL-4, IL-13, or STAT-6 do not have elevated levels of Relm-*α* following an immune challenge [156]. Thus, Relm-*α* appears to serve as a negative feedback loop regulating the magnitude of host response to helminth infection. Besides, Relm-*α* appeared to be a mediator of tissue repair as the absence of AAM-derived Relm-*α* in skin wounds of *Lyz2^cre^Il4ra^−/lox^* mice was associated with a failure of the healing response [157].

Programme death ligand 2 (PD-L2) is upregulated in monocyte-derived AAMs, whereas it is poorly expressed on IL-4-treated resident macrophages [158]. PD-L2 is a ligand of PD-1, a potent inhibitory receptor expressed on effector T cells, and macrophage PD-L2 has been shown to potently inhibit T cell proliferation [159]. PD-L2 expression is upregulated in lung macrophages after *N. brasiliensis* infection whereas STAT-6-deficient mice display low levels of macrophage PD-L2 [159]. In addition, *in vivo* blockade of PD-L2 during *N. brasiliensis* infection increases type 2 cell-mediated cytokine responses in the lung, further indicating that AAMs inhibit Th2 cells by expression of PD-L2. In addition to its effector function, PD-L2 is used as a relevant surface marker to distinguish between resident and monocyte-derived macrophages following antigenic stimulation or *S. mansoni* infection [125, 127]. Differences in the dynamics of macrophage responses between mouse strains could explain variation in susceptibility to helminth infections. Campbell and colleagues showed that macrophage responses against *L. sigmodontis* infection in susceptible BALB/c mice was dominated by infiltration of monocytes with the immunosuppressive PD-L2^+^ phenotype, as opposed to resistant C57BL/6 mice in which expansion of resident cells was the main source of macrophages [117].

## 4. Conclusion and Future Directions

Parasitic helminths share many aspects as they all induce strong type 2 cell-mediated immune responses. However, they are not completely equal in terms of the immune responses they induce and the ability of the mounted immunity to deal with effective clearance of the parasite, avoid immunopathology, and/or promote tolerance of chronic low-burden infection. Powerful tools have been used to study macrophage origin, polarization, activation, and function after helminth infection, and yet, many questions are still unresolved. What makes the difference between filarial nematodes inducing local macrophage proliferation via IL-4 and *S. mansoni* eggs promoting monocyte recruitment? What functions are mediated by AAMs during acute schistosomiasis where conflicting results arise from the use of *Lyz2^cre^Il4ra^−/lox^* mice? Would new gene identification from single-cell sequencing on macrophage populations in the various tissues affected by helminth infection such as the skin, lung, liver, or intestine and depending on given time points following infection ultimately permit the development of novel conditional knockdown mouse models to provide clearer information on the role of such plural macrophage responses? What are the interactions between liver resident macrophages, granuloma-derived macrophages after *S. mansoni* infection, and hepatic stellate cells in the regulation of fibrosis? Although there is no doubt that our understanding is still limited on how the host immune system and macrophage responses have been shaped during evolution to deal with parasitic helminths and whether we can truly consider them as “old friends,” we are convinced that new insights will be acquired in the near future to resolve these questions and others.

## Figures and Tables

**Table 1:** Common and potential mouse strains and reagents to examine AAM functions during parasitic helminth infection.

Methodology	Effect	Limitation (major phenotype after helminth infection)	References
Deletion of phagocytes			
Clodronate liposomes	Depletion of circulating phagocytes if intravenous administration	Difficult-to-control efficacy (reduced survival after *S. haematobium* infection, defect in *H. polygyrus* control)	[160]

Diphtheria toxin treatment			
CD11b-DTR	Specific depletion of macrophages, monocytes, and granulocytes	Nonspecific	[145]
CCR2-DTR	Depletion of Ccr2^+^ circulating monocytes	CCR2-independent monocyte functions cannot be investigated (severe acute weight loss in response to *S. mansoni* infection)	[88]
CX_3_CR_1_-DTR	Depletion of CX_3_CR_1_^+^-patrolling monocytes	Not specific to patrolling monocytes	[161]
CD169-DTR	Specific depletion of CD169^+^ cells	Target the majority of resident tissue macrophages in many tissues	[162]
Clec4F-DTR	Specific depletion of Küpffer cells	Induce major monocyte recruitment in the liver	[119]

Neutralizing or depleting antibodies			
Anti-CCR2 Ab (clone MC-21)	Depletion of Ccr2^+^ circulating monocytes	CCR2-independent monocyte functions cannot be investigated	[163]
Ym1 neutralizing antibodies	Ym1 blockade	Not restricted to macrophages (increased neutrophil infiltration and IL-17 production by *γδ* T cells)	[151]

Knockouts			
*Il4ra^−/−^*	Global defect in IL-4 and IL-13 signaling	Not restricted to macrophages (defect in nematode control and increased susceptibility to *S. mansoni*)	[54] [86]
*Arg1^−/−^*	Global defect in arginase 1	Not restricted to macrophages (increased immunopathology in BM chimeras to *S. mansoni*)	[144]
*Mrc1^−/−^*	Global defect in CD206 (mannose receptor)	Not restricted to macrophages	[133] [154]
*Retnla^−/−^*	Global defect in Relm-*α*	Not restricted to macrophages (increased type 2 cell-mediated cytokine responses after helminth infection)	[58]
*Chil1^−/−^*	Global defect in *BRP-39*	Not restricted to macrophages (increased IL-17 production by *γδ* T cells)	[151]
*Chia1^−/−^*	Global defect in *AMCase*	Not restricted to macrophages (defect in the control of gastrointestinal nematodes)	[149]

Conditional knockdowns			
*Lyz2^cre^Il4ra^−/lox^*	Defect of IL-4 and IL-13 signaling in macrophages and neutrophils	Not specific to macrophages IL-4R*α* expression retained in immature *Lyz2^low^* macrophages (no effect on nematode infection and conflicting results after *S. mansoni* infection)	[54] [80] [121] [86]
*Lyz2^cre^Arg1^lox/lox^*	Knockdown of arginase 1 in macrophages and neutrophils	Not specific to macrophages Arg1 expression retained in immature *Lyz2^low^* macrophages? (increased type 2 immunity and collagen deposition in chronic schistosomiasis)	[40]
*Tie2^cre^Arg1^lox/lox^*	Knockdown of arginase 1 in hematopoietic and endothelial cells	Not specific to macrophages but Arg1—mainly restricted to macrophages and hepatocytes (increased type 2 immunity and collagen deposition in chronic schistosomiasis)	[40]
